# Breast and cervical cancer screening among women in metropolitan areas of the United States by county-level commuting time to work and use of public transportation, 2004 and 2006

**DOI:** 10.1186/1471-2458-10-146

**Published:** 2010-03-19

**Authors:** Steven S Coughlin, Jessica King

**Affiliations:** 1 Division of Cancer Prevention and Control, National Center for Chronic Disease Prevention and Health Promotion, Atlanta, GA, USA; 2 Environmental Epidemiology Service, Department of Veterans Affairs, Washington, DC, USA

## Abstract

**Background:**

Commuting times and behaviors have been associated with a variety of chronic disease outcomes and health behaviors. We examined the relationships between ecologic measures of commuting time and use of public transportation in relation to breast and cervical cancer screening among women in U.S. metropolitan areas who participated in the 2004 and 2006 Behavioral Risk Factor Surveillance System (BRFSS) surveys.

**Methods:**

Self-reported county of residence was used to classify respondents as residents of metropolitan statistical areas (MSAs). Only BRFSS respondents who resided in the 39 MSAs with a population of ≥ 1.5 million in 2007--representing a total of 337 counties--were included in this analysis. A total of 76,453 women aged ≥ 40 years were included in analyses on mammography. Analyses on Pap testing were limited to women aged ≥18 years with no history of hysterectomy (n = 80,959). Area-based measures of socio-economic status (SES) were obtained by utilizing county-level information from the 2000 U.S. Census.

**Results:**

With adjustment for age, no important associations were observed between receipt of a recent mammogram and either a county-level measure of commute time or residence in an area where more residents had access to a car. Similarly, women living in counties where at least four percent of the residents used public transportation were as likely to have had a recent mammogram or Pap test compared with women in areas where less than four percent of residents used public transportation. However, women living in counties where < 2% of residents had no access to a car were somewhat more likely to have had a Pap test in the past 3 years than women in areas where ≥ 3% of the residents had no access to a car (87.3% versus 84.5%; p-value for test for trend < 0.01). In multivariate analysis, living in a county with a median commute time of at least 30 minutes was not significantly associated with having had a Pap test in the past 3 years (adjusted odds ratio (OR) = 1.1, 95% CI 0.9-1.2, p = .50), or with having had a mammogram in the past 2 years (adjusted OR = 0.9, 95% CI 0.9-1.1, p = .28). A weak positive association was observed between residence in a county with less use of public transportation and having had a Pap test in the past 3 years, which was of borderline significance (adjusted OR 1.2, 95% CI 1.0-1.4, p = .05).

**Conclusions:**

In large U.S. metropolitan areas, transportation issues may play a role in whether a woman obtains cancer screening along with other factors (e.g., Hispanic ethnicity, low income, and no physician visit in the past year). In this contextual analysis, a longer commute time was not associated with breast and cervical cancer screening.

## Background

Screening mammography is currently the most effective method of detecting breast cancer early and reducing mortality from the disease. Routine use of the Papanicoloau (Pap) test has been associated with reduced incidence of cervical cancer. A number of studies have identified socio-demographic and health systems-related characteristics that are barriers or facilitators of breast and cervical cancer screening [1-7]. Well-established barriers to screening include individual characteristics such as lower income, lower educational attainment, and lack of health care insurance and factors related to the health care system such as lack of a recommendation for screening by a health care provider and lack of patient or provider reminder systems [1-7]. Ecological factors related to access to health care (for example, socioeconomic and demographic characteristics of the neighborhoods in which women live) have also been associated with use of breast and cervical cancer screening [1,8-10].

Studies have examined cancer screening and treatment outcomes in relation to the distance that women have to travel to get to screening mammography or treatment facilities [11-17]. A study of Medicare beneficiaries aged 65 to 79 in Kansas, a predominately rural state, found that mammography utilization was somewhat higher among residents of counties with permanent mammography facilities, but that geographic variation in mammography rates was not well-explained by proximity to mammography facilities [13]. A study of patients with stage I or II breast cancer, identified using data from the Surveillance, Epidemiology, and End Results (SEER) registry, found that women residing an increased distance from a hospital with a radiotherapy facility had a decreased likelihood of undergoing breast-conserving surgery [16]. Similar results were found in studies of female breast cancer patients in New Mexico and in rural areas of Michigan and New Hampshire [11,12,15]. To date, most studies of distance traveled to cancer screening or treatment facilities have been carried out in rural populations. Rural areas differ from metropolitan areas in a number of factors potentially associated with use of preventive services including commuting time and use of public transportation.

Commuting times and behaviors have been associated with a variety of chronic disease outcomes and health behaviors including obesity [18,19], decreased physical activity [20], and cardiovascular disease [21]. Traffic congestion, as many residents of large metropolitan areas are keenly aware, is characterized by slower automobile and bus speeds and longer trip times [22,23]. In many U.S. cities, more severe traffic congestion leads to traffic jams, resulting in delays in commuting and wasted time and resources. In many instances, traffic congestion and increased commuting time are potential problems even for persons who use public transportation (e.g., those who rely upon city buses to get to work or to a health care provider).

The objective of this study was to examine the relationships between ecologic measures of commuting time, use of public transportation, and access to an automobile in relation to breast and cervical cancer screening, among women in U.S. metropolitan areas who participated in the 2004 and 2006 Behavioral Risk Factor Surveillance System (BRFSS) surveys. Although ecological measures of commuting time and use of public transportation were primarily used as surrogates of individual-level measures of commuting time and use of public transportation, we also considered the possibility that ecologic measures of commuting time, use of public transportation, and access to an automobile might also be associated with other unmeasured characteristics of metropolitan areas. We hypothesized a priori that women who live in metropolitan areas with longer commuting times would be less likely to adhere with guidelines for breast and cervical cancer screening, since more of their daily routine is spent commuting and we thought it plausible that they may spend more of their financial resources on commuting costs. However, in view of the sparse literature on this topic, we considered this to be a two-sided hypothesis (i.e., we did not have an a priori opinion about the direction of the association).

## Methods

The data used in the current study were obtained from the 2004 and 2006 Behavioral Risk Factor Surveillance System (BRFSS), a state-based system of telephone health surveys. Data from 2004 and 2006 were combined in order to increase the sample available for analysis. The BRFSS uses a random-digit dialing technique and multistage cluster sampling in each participating state in order to sample non-institutionalized adults living in a residence that had a telephone [24]. Trained interviewers administered the computer-assisted telephone interviews.

The current study sample was drawn from women aged 18 years or older who responded to BRFSS surveys in 50 states and the District of Columbia. All eligible women were included regardless of their self-identified race and Hispanic ethnicity. For the study outcome of having had a Pap test within the previous three years, the eligible sample consisted of women who reported a known Pap test screening status, were aged 18 years or older, and who reported no history of a hysterectomy (n = 80,959). For the study outcome of having had a mammogram within the previous two years, the eligible sample consisted of women who reported a known mammography screening status and were aged 40 years or older (n = 76,453).

Self-reported county of residence was used to classify respondents as residents of metropolitan statistical areas using Office of Management and Budget (OMB) definitions, revised in November 2007. All counties within the metropolitan areas were included regardless of state boundaries. In order to ensure there were sufficient numbers of respondents in each metropolitan area, only BRFSS respondents who resided in Metropolitan Statistical Areas (MSAs) with a population of at least 1.5 million in 2007 were included in this analysis (Additional file 1, Figure 1).

These account for about 27% of female BRFSS respondents during this time period. The 39 MSAs included ranged in population size from 18,815,988 to 1,521,437 persons.

County-level measures of commuting time within Metropolitan Statistical Areas (MSAs) (< 30 minutes vs. ≥ 30 minutes), use of public transportation (<4% vs. ≥ to 4%, where public transportation included use of a bus or trolley bus, streetcar or trolley car, subway or elevated railway, railroad, ferryboat, or taxicab), and automobile access (≤ 1%, 2%, or 3%+ with no access to car) were obtained from 2000 Census data, summary file 3 [25]. Variables used included: means of transportation to work for workers 16 years and over (table P30), tenure by vehicles available (table H44), travel time to work for workers 16 years and over (table P31), and tenure by vehicles available (table H44). The categories for commuting time at the county level used in our analysis were constrained by the way in which the data are presented by the US Census Bureau. County-level data available from Census 2000 on travel time to work are categorized as: less than 5 minutes, 5 to 9 minutes, 10 to 14 minutes, 15 to 19 minutes, 20 to 24 minutes, and so forth, all the way up to 90 minutes or more. We collapsed these time categories into <30 minutes versus > = 30 minutes and calculated the percent of county residents reporting commute times within those 2 broad categories. We then obtained the median percentages by county and categorized this variable as "At least MSA median % have commute greater than or equal to 30 minutes" versus "Less than MSA median % have commute greater than or equal to 30 minutes.

Other county-level socio-economic status indicators used were sex-specific educational attainment for the population 25+ years (table P37), sex-specific employment status for the population 16+ years (table P43), and poverty status in 1999 by age (table P87).

To account for the complex sample design of the BRFSS, all cancer screening percentages were weighted and standard errors calculated using SUDAAN statistical software [26]. Weights were used to adjust for differences in probability of selection, non-response, and non-coverage. The estimated median response rates were 41.2% for 2004 and 35.4% for 2006 [24]. The study included BRFSS questions about general health status, demographic and socioeconomic factors, access to health services, mammography, and Pap testing.

In addition, except for age group-specific estimates, percentages were age-adjusted to the July 1, 2000 female population using United States Census estimates. Self-reported Pap test and mammography rates were estimated by various demographic, socioeconomic, or health-related individual-level covariates, and by county-level measures of commuting time, use of public transportation, automobile access, and employment status. The Cochran-Mantel-Haenszel test of association was used to assess the overall statistical significance of each potential factor, such as the association of health insurance status with cancer screening test use, after adjusting for age. A multivariate analysis of correlates of screening test use was conducted using logistic regression techniques and SUDAAN, to take the weighting and complex sampling into account, and to further adjust for covariates found to be related to cancer screening rates, according to ecologic measures of commuting times and traffic congestion. Marital status was omitted from the multivariate models because of the weak associations with cancer screening. Adjusted Wald F tests were used to determine the significance of the model and for selection of variables.

The data used in this analysis are anonymous and included no personally identifying information. This analysis of existing data from an anonymous survey (BRFSS) combined with US Census Data at the county level was determined to be exempt from institutional review board review according to Centers for Disease Control and Prevention guidelines.

## Results

Characteristics of the 81,820 women who were at least 18 years of age at time of the survey administration and reported no history of hysterectomy are shown in Additional file 2, Table 1.

Over 10 percent of the women had less than a high school education. About 10 percent had a household income of less than $15,000 per year. Almost 15 percent had no health insurance. About 37 percent of the women lived in counties where at least 13 percent of residents lived below the poverty level. Over half of the women lived in counties where at least four percent of residents used public transportation.

Rates of self-reported mammogram in the past two years are shown in Additional file 2, Table 2a by individual-level demographic and socioeconomic variables, variables associated with health care access, and area-based measures of use of public transportation and commuting time. With adjustment for age, no important associations were observed between receipt of a recent mammogram and with either residence in a county with a median commute time of over 30 minutes or residence in an area where more residents had access to a car. Similarly, women living in counties where at least four percent of the residents used public transportation were as likely to have had a recent mammogram as women in areas where less than four percent of residents used public transportation.

Rates of Pap testing in the past three years are shown in Additional file 2, Table 2b by individual-level demographic and socioeconomic variables, variables associated with health care access, and area-based measures of use of public transportation and commuting time. With adjustment for age, no important associations were observed between receipt of a recent Pap test and with either residence in a county with a median commute time of over 30 minutes or use of public transportation. However, women living in counties where < 2% of residents had no access to a car were somewhat more likely to have had a Pap test in the past 3 years than women in areas where ≥ 3% of the residents had no access to a car (87.3% versus 84.5%; p-value for test for trend < 0.01).

In multivariate analysis (Additional file 2, Table 3), controlling for calendar year, age, race, Hispanic ethnicity, education, household income, check-up by a physician in the past year, health care coverage, general health status, and county-level variables such as percentage living below the poverty level, percentage with less than a high school diploma, percentage unemployed, and percentage use of public transportation, residence in a county with a median commute time of at least 30 minutes was not associated with having had a mammogram in the past 2 years (adjusted odds ratio (OR) = 0.9, 95% CI 0.9-1.1, p = .28). No significant association was observed with percentage use of public transportation. In a similar model for recent Pap test, residence in a county with a median commute time of at least 30 minutes was not associated with having had a Pap test in the past 3 years (adjusted odds ratio (OR) = 1.1, 95% CI 0.9-1.2, p = .50). A weak association was observed between residence in a county with less use of public transportation and having had a Pap test in the past 3 years, which was of borderline significance (adjusted OR = 1.2, 95% CI 1.0-1.4, p = .05).

## Discussion

The findings of this study contribute to the growing literature on socioeconomic and demographic characteristics of the neighborhoods in which women live that are associated with use of breast and cervical cancer screening [1,8-10]. With adjustment for individual-level factors such as age, race, Hispanic ethnicity, health insurance, and recent physician visit, and a variety of county-level variables associated with socioeconomic status and access to health care, women living in a county with a median commute time of at least 30 minutes were as likely to have had a recent Pap test or mammogram test as those living in counties with shorter commute times. This was contrary to our a priori hypothesis that women with longer commutes would be less likely to get screened. With adjustment only for age, no important associations were observed between receipt of a recent mammogram or Pap test and commuting variables, with the exception that women living in counties where < 2% of residents had no access to a car were somewhat more likely to have had a Pap test in the past 3 years compared with women in areas where ≥ 3% of the residents had no access to a car (p-value for test for trend < 0.01). These apparent differences according to whether adjustment was made only for age or for several predictors of breast and cervical cancer screening are likely to be partly accounted for by confounding of age-adjusted results by race, Hispanic ethnicity, and socioeconomic factors. Ethnic and racial differences in socioeconomic status and commuting patterns are well-documented. In Census 2000, a much higher proportion of non-Hispanic white workers drove alone to work than workers of other races or Hispanic origin [22]. Hispanic workers were least likely to drive alone to work. People who were non-Hispanic white were least likely to take public transportation or to carpool [22].

We cannot rule out the possibility that the weak association observed in multivariate analysis between residence in a county with less use of public transportation and having had a Pap test in the past 3 years may be accounted for by uncontrolled confounding by unmeasured individual-level variables or county-level characteristics. For example, BRFSS data do not included information about health literacy, knowledge about cancer, or attitudes toward routine cancer screening.

Penchansky and Thomas [27] described five potential dimensions to categorize access to health care including availability (related to volume of services), accessibility (described as travel distance or impedance), accommodation (related to convenience), affordability, and acceptability (related to characteristics of providers). Much of the research and policy efforts around access to care and health care disparities have focused on affordability issues such as health care expenditures, reimbursement, out-of-pocket expenses, or health care insurance. Less is known about geographical availability and accessibility, especially for urban areas [28,29]. The literature on spatial disadvantage and geographical distance as a barrier to cancer screening and access to medical care has often focused on rural areas [12,13,15]. In contrast with rural areas, distances to facilities in urban areas are shorter and multiple transportation pathways are available for residents. Nevertheless, spatial accessibility in urban areas can still pose a challenge, especially for minorities and low-income urban residents who might be more likely to depend on public transportation. For example, among African-American households in Atlanta, more than 15% do not have access to a private vehicle [22]. Among whites, less than 4% do not have access to a private vehicle [22]. More recently, a study of the spatial distribution of Chicago's low- or no-cost mammography screening facilities showed overall shorter travel time for low income residents but longer travel time and distances from African American neighborhoods than for other lower-income neighborhoods [17]. A related factor, convenience of appointment time and location, has also been shown to negatively impact mammography screening [30,31]. Economic research has demonstrated that there is a spatial mismatch between dispersed urban employment opportunities and residential locations that is exacerbated by public transportation systems that fail to reach these areas [32].

The ability of this study to identify specific pathways influencing screening utilization was limited by the variables included in the analytic data set, by the smallest geographic area of residence available for analysis (county), and by the ecological nature of the study. A further issue is that the effects of transportation variables on breast or cervical cancer screening may vary across large metropolitan areas. In the U.S., transportation infrastructure, facilities, and utilization patterns vary across major metropolitan areas. For example, not all cities have low-cost subway systems or trolley cars. In Census 2000, public transportation use was concentrated in the Northeast, and carpooling was concentrated in the South and the West [22]. As previously mentioned, there may have been uncontrolled confounding due to unmeasured individual- or county-level variables. With respect to other limitations of the current study, no information was available about the work locations of the women or the proximity of cancer screening facilities to place of employment. The current study did not examine data on mobile screening units. A 2006 report by the Government Accounting Office indicated that there were 222 mobile mammography facilities in the United States. A previous study [33] found that the use of mobile units in the United States was limited. Of 1,057 mammography facilities, 2.4% were identified as mobile and accounted for 3% of mammography examinations performed [33]. In the current study, response bias is also a possibility since many potential participants did not participate and the BRFSS excludes women living in households without telephones. Self-reported information about cancer screening may also differ from information obtained from records of health care providers [34,35].

## Conclusions

By way of summary, in this contextual analysis, select commuting variables were not significantly associated with higher breast and cervical cancer screening. Living in a county with less use of public transportation was weakly associated with cervical cancer screening but the association was of borderline significance. In large metropolitan areas, transportation issues play a role in whether a woman obtains cancer screening along with other factors (for example, Hispanic ethnicity, low income, and no physician visit in the past year). Future contextual analyses of the utilization of cancer screening services within specific metropolitan areas could include additional transportation and commuting variables and examine neighborhood characteristics in greater detail at the census tract level or block group level.

## Competing interests

The authors declare that they have no competing interests.

## Authors' contributions

Both authors made substantial contributions to the conception and design of the study and interpretation of the data. SC drafted the manuscript. JK analyzed the data and reviewed the manuscript and tables for accuracy. Both authors read and approved the final manuscript.

## Pre-publication history

The pre-publication history for this paper can be accessed here:

http://www.biomedcentral.com/1471-2458/10/146/prepub

## Supplementary Material

Additional file 1 Excel file containing figure 1. Click here for file

Additional file 2 Excel file containing table 1, table 2a and 2b, and table 3. Click here for file
